# Bioethics Artificial Intelligence Advisory (BAIA): An Agentic Artificial Intelligence (AI) Framework for Bioethical Clinical Decision Support

**DOI:** 10.7759/cureus.80494

**Published:** 2025-03-12

**Authors:** Taposh P Dutta Roy

**Affiliations:** 1 Responsible AI, Kaiser Permanente, Oakland, USA; 2 Bioethics, Harvard Medical School, Boston, USA

**Keywords:** agentic ai, ai, ai bioethics, bioethics framework, bioethics recommendations

## Abstract

Healthcare professionals face complex ethical dilemmas in clinical settings in cases involving end-of-life care, informed consent, and surrogate decision-making. These nuanced situations often lead to moral distress among care providers. This paper introduces the Bioethics Artificial Intelligence Advisory (BAIA) framework, a novel and innovative approach that leverages artificial intelligence (AI) to support clinical ethical decision-making. The BAIA framework integrates multiple bioethical approaches, including principlism, casuistry, and narrative ethics, with advanced AI capabilities to provide comprehensive decision support. The framework employs a structured methodology that includes data collection, paradigmatic case review, analysis through "mattering maps," and scenario-based decision reasoning. A detailed analysis of two challenging cases, an end-of-life care decision and a complex conjoined twins case, demonstrates BAIA's potential to harmonize diverse ethical perspectives while reducing the moral burden on healthcare providers. The framework's agentic architecture additionally allows integration with any new and existing ethical AI systems like METHAD, Delphi, and EAIFT, enabling multiframework collaboration. This work also acknowledges limitations related to data quality, bias, and complexity of ethical decisions and proposes mitigation strategies, including standardized databases, fairness algorithms, and maintaining human oversight. Thus, this work represents a significant step toward combining technological advancement in agentic AI with established bioethical principles to improve the quality and consistency of clinical ethical decision-making, thus reducing moral distress for clinicians.

## Introduction

The integration of analytics in healthcare traces back to 1854 when Dr. John Snow [1] first illustrated the use of systematic data analysis to mark the end of cholera in London. In the following 170 years, significant advances have emerged in medicine and computer science. While technology advances, clinical decision-making remains particularly challenging when healthcare teams face ambiguous, emotional, and complex decisions involving end-of-life care, informed consent [2], surrogate decision-making [3], genetics [4], futility [5], harm principle [6], and others. These decisions impact the care team and lead to moral distress [7], residue [8], and injury. The potential of artificial intelligence (AI) to serve as an advisor to support decision-making [9] and reduce moral impact can significantly benefit the team, patient, and family. This essay proposes an innovative Bioethics Artificial Intelligence Advisory (BAIA) framework to augment human reasoning in clinical decision-making. BAIA complements healthcare teams in navigating complex ethical dilemmas by integrating bioethical approaches, including principlism, casuistry, narrative ethics, and agentic AI capabilities. Through the analysis of two challenging cases, an end-of-life care decision and a complex conjoined twin’s case, this framework demonstrates its potential to harmonize diverse ethical perspectives, reduce moral distress and moral burden on the care providers, and enhance the quality and consistency of decisions in highly complex and emotional clinical environments.

## Technical report

Agentic AI system 

**Figure 1:**
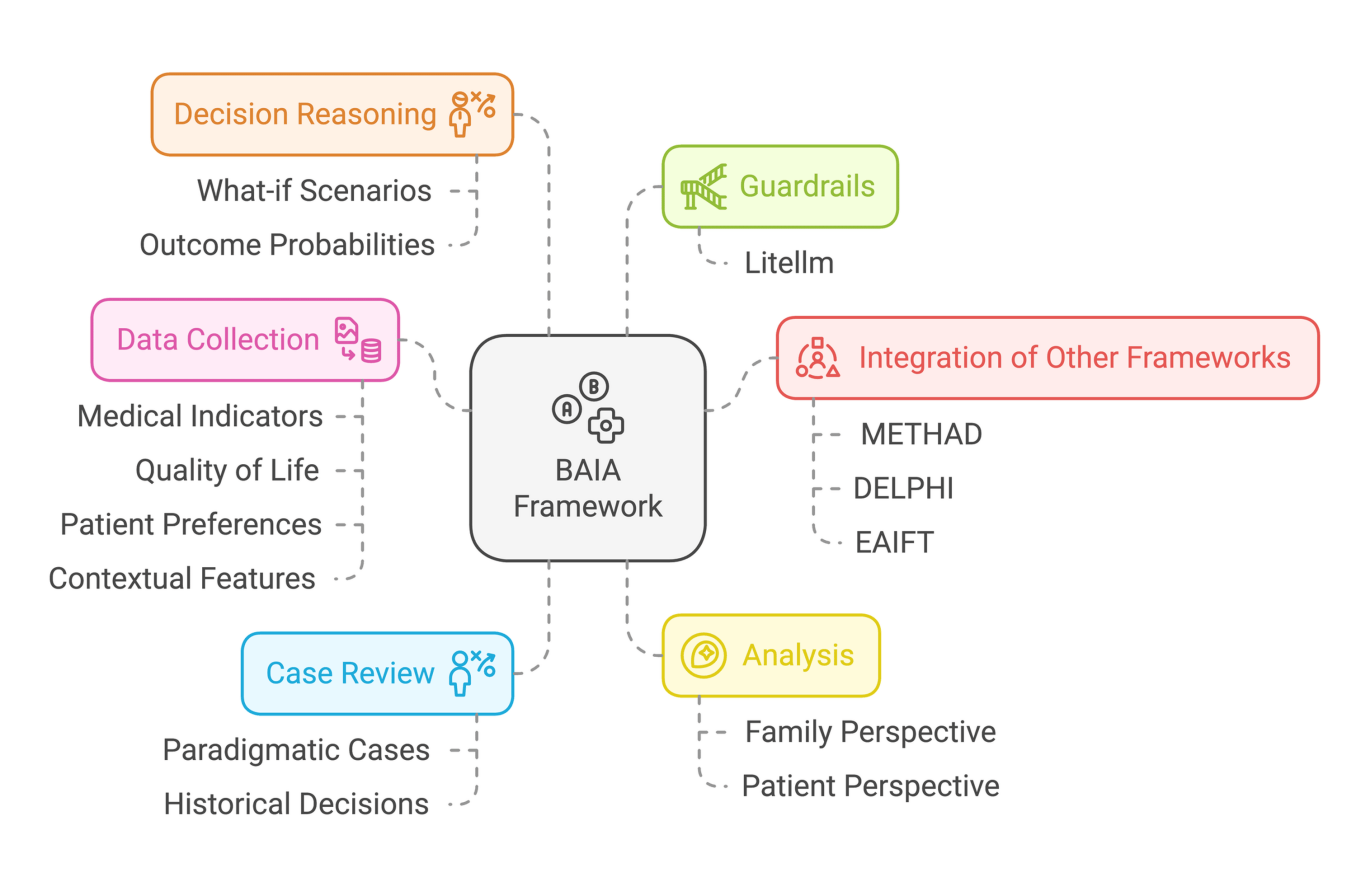
Components and capabilities of the BAIA framework BAIA: Bioethics Artificial Intelligence Advisory Figure credits: Taposh Dutta Roy, image created using napkin.ai

An AI system is trained on a large amount of data and learns statistical patterns to predict the next word in a sequence [10]. When enhanced with the capability of invoking other programs, these are called “Agents”. Chawla et al. [11] define “Agentic AI” as a framework in which large language models enable workflows, supporting four capabilities: tool usage for accuracy enhancement using external sources, self-correction, structured task breakdown, and multi-model collaboration. As of this writing, there are three distinct AI systems for ethical decision-making: DELPHI [12], Medical Ethics Advisor (METHAD) [13], and Ethical Artificial Intelligence Framework Theory (EAIFT) [14]. EAIFT embeds ethical reasoning within AI systems to guarantee their ethical operation. DELPHI is a framework for moral reasoning, leveraging AI to determine ethically acceptable actions. METHAD focuses on clinical ethics dilemmas and models Beauchamp and Childress’s (B&C) [15] autonomy, beneficence, and nonmaleficence utilizing fuzzy cognitive maps (FCMs) [16], a method for modeling cause-and-effect relationships and interconnected concepts. However, METHAD misses the concrete case-specific details and narrative approaches [17], which add context to the family and patient’s perspective. Each of the above systems uses a different methodology, with its strengths and weaknesses, and investigates different sides of ethical AI (Figure 1). 

BAIA framework

**Figure 2:**
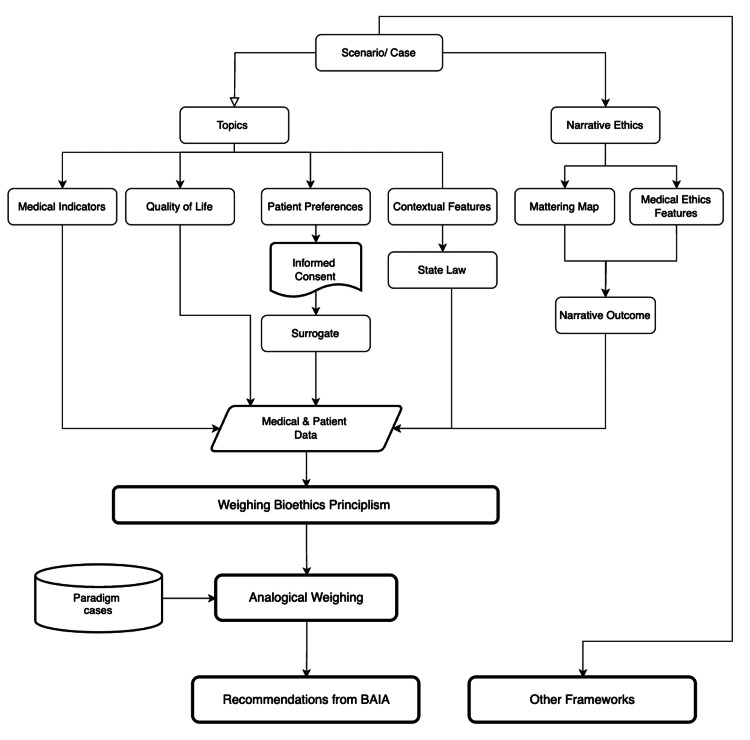
BAIA framework details BAIA: Bioethics Artificial Intelligence Advisory Figure credits: Taposh Dutta Roy, image created using the flowcharting tool draw.io

Today, clinical ethics teams use principlism as outlined by B&C to support complex, time-sensitive, and strenuous healthcare decisions. B&C’s principlism [15] has stood the test of time and provides a robust yet abstract approach to ethical decision-making. Other moral theories, such as casuistry [18] and narrative ethics [17], provide case-level details and storytelling to make the decision-making approach concrete. Current frameworks such as METHAD follow principlism, DELPHI leverages AI for moral reasoning, and EAIFT embeds ethical decision-making in AI. This work proposes BAIA, a novel framework developed in response to the limitations of existing AI-driven ethical decision-making tools. BAIA uses a scaleable agentic AI strategy that incorporates B&C’s principlism [15], casuistry [18], and narrative ethics [17]. BAIA expands casuistry’s first step, “topics or case container,” [18] to collect data for medical indicators, quality of life, patient preferences, and contextual features by adding features from narrative ethics such as storytelling and extracting data on voice, character, plot, and resolution [17]. Next, we review the paradigmatic [18] cases so we can learn from past decisions. A paradigmatic case review is part of casuistry, where one reviews a past case similar to the case in hand to get a historical perspective. The third step is "analysis," developing “mattering maps [19],” a narrative ethics concept used for the representation of the family and patient’s perspective of what is most important in their life and how they got to this point. It also weighs B&C’s principles based on the data available from prior steps. The fourth step is decision reasoning, where, based on the information, the system develops “what-if” capability for the scenarios and their probability of outcomes. Additional methods and theories, such as deontology, utilitarianism, etc., can be added to the final step to incorporate different viewpoints. The BAIA becomes one agent in our agentic strategy, while METHAD, DELPHI, and EAIFT become other decision-making agents. As more frameworks evolve, our agentic system can easily be expanded to incorporate newer details. Additionally, we define guardrails such as bias and drift detection, human-in-the-loop oversight, and explainability mechanisms for the agentic framework utilizing the open-source LiteLLM [20]. With BAIA's multimodel agentic capability, we can review clinical cases and provide advisory data points (Figure 2).

## Discussion

Case analysis 

Case 1: End-of-Life Care

Consider the case of a 68-year-old male patient [21] with severe impairments, myocardial infarction, stroke, hemiplegia, and multiple organ failure. His family insisted on “full code,” including aggressive life-prolonging interventions such as cardiopulmonary resuscitation (CPR) to save his life, despite the physician’s view that these may be futile. The hospital requested the Court of Protection to withhold CPR, invasive hemodynamic support, and renal replacement therapy in the event of future degradation, which was rejected. Applying the proposed BAIA framework in this situation, the ethics team gathers topical data, such as medical indicators, quality of life, patient preferences, and contextual features, and conducts narrative interviews with family members, physicians, and nursing leaders to understand the voice, character, plot, and resolution. The BAIA framework reveals the family's emotional motivations and cultural beliefs through these interviews. Next, the framework will look for a similar case from the past; if one is found, it will become the "paradigmatic" case utilized in this context. The system analyzes two key points. First, it creates “mattering maps” that highlight the moral weight of prolonging life versus alleviating suffering from the perspectives of both patient and family. Second, it evaluates the principles of beneficence and nonmaleficence to develop a balanced scorecard, and finally, it formulates a “what-if” analysis, which is a simulation capability that provides outcomes and explanations through scenario modeling. For example, one scenario could involve discontinuing futile interventions and transitioning to palliative care, while another might consider continuing with “full code” treatment. The patient's family insisted on doing everything possible to save him. This situation falls under “positive rights,” where patients have the right to receive medical care but do not have the right to interventions that exceed appropriate medical care. The BAIA framework will take into account the family’s perspective along with all case details, such as topical data and paradigm cases. This structured process ensures that the family's voice is acknowledged while adhering to the ethical principles of beneficence and nonmaleficence. Additionally, the BAIA framework will also seek guidance from other systems such as METHAD and DELPHI. The BAIA framework synthesizes all information to provide recommendations and the potential to run additional scenarios and consult other frameworks or approaches. Utilizing this AI framework would reduce moral distress, enhance quality, and bring consistency to decision-making for the patient.

Case 2: Conjoined Twins

Cummings et al. published a case report about 22-month-old conjoined twins ( “Twin A” and “Twin B”) [22], highlighting the tension between medical possibility and ethical boundaries. The twins were born in East Africa and arrived at Massachusetts General Hospital for evaluation of separation. They shared a single liver, an abdominal cavity, and a portion of their gastrointestinal tract. Twin B was larger and healthier, while Twin A had complex congenital heart disease and relied on her sibling’s circulation for support. Unfortunately, Twin A’s condition worsened, which required the twins to be admitted to the pediatric intensive care unit for stabilization and treatment. Applying our proposed BAIA framework to this case, specific medical, quality of life, and contextual information such as Islamic religion and advice from their local Imam are obtained. Since the patients are pediatric, parental consent was a necessity for any intervention. The ethics team conducts narrative interviews with parents and other care providers. Given the rarity of conjoined twins, we may not find a good paradigmatic case. The system will develop from the parent’s perspective a “mattering map” and analyze the case considering various concepts such as beneficence, nonmaleficence, the doctrine of double effect [23,24], pediatric informed consent, self-driven car facing a choice between hitting someone on a crosswalk or killing themselves, etc. The decision-reasoning step will provide a recommendation and the ability to do a scenario analysis considering various possibilities. The BAIA framework will evaluate various perspectives [22], including each twin’s likelihood of survival, the parents' religious beliefs, and refusal of surgery. Additionally, it will take into account the doctrine of double effect, which recognizes the intention to act in the best interest while acknowledging that “Twin A” may not survive, effectively designating her as a “marked for death” patient [22]. BAIA will provide an advisory recommendation and explanation that respects the family's values, thus reducing the moral distress and providing consistent decisions for the case.

BAIA strengths

Using the two cases, we show that the proposed BAIA framework provides a comprehensive and structured approach to making complex treatment decisions. It utilizes data from the case, narrative stories, and a principled approach. The framework’s reliance on data collection ensures that all pertinent information, such as medical information, quality of life, patient preferences, contextual information, and narrative stories, is collected. Incorporating a paradigmatic case ensures that we draw insights from similar scenarios in the past. In the analysis phase, we develop “mattering maps” [17], a patient perspective on “how they got here” and what their wishes are, adding depth and developing a human context. Further, the ability to do scenario analysis provides ways to plan the situation and weigh the pros and cons of each. Compared to existing frameworks METHAD [13] and DELPHI [12], BAIA provides concrete case-specific depth, reasoning, and a data-driven approach. Finally, using an agentic technology makes the BAIA framework expandable and additive to any new approach.

BAIA opportunities

Despite its comprehensive approach, BAIA has several limitations. First, its analysis relies on high-quality, unbiased, and comprehensive datasets, which can be challenging due to access issues or incomplete data capture. Second, the outcomes of the BAIA algorithm must be appropriate, fair, and unbiased. Validating these outcomes is increasingly important for BAIA, as it can be complex to determine the correct answer. Third, ethical decisions are multifaceted and nuanced, which AI systems might oversimplify. We should set up the following tools and strategies to mitigate these limitations. First, develop a standardized database with diverse anonymized cases. These cases should be revisited for validation and appropriately tagged if they contribute to any decision-making. Second, fairness and bias [25] detection algorithms should be established to validate the outcomes. The validation strategy should include model outcome explanation methods such as SHAPley Additive exPlanations (SHAP) [26], causality [27,28], and counterfactual [29] analysis. Furthermore, every outcome report should contain a probability of consideration and a reasoning-based chain of thought [30] informing the decision recommendation. Additionally, a “human in the loop [31]” approach will ensure that care professionals remain central to the decision-making process. Finally, the time and resources required to use the framework could limit its feasibility in time-sensitive situations. Addressing these limitations, including a standardized database of anonymized cases, data fairness, bias detection, explainable outcomes, and “humans in the loop,” will enhance BAIA’s ability to support complex decisions while upholding human values.

## Conclusions

Safeguarding patient well-being and preserving human values are at the heart of healthcare. This theoretical approach utilizes the latest technological advancements, such as large language models and agentic AI, to develop a solution for nuanced real-world problems. It builds on the work done by prior scholars and develops a comprehensive system that looks at abstract bioethical principles and case-specific details to provide advisory support. Further, the ability to extend the framework to existing developed methods makes it flexible to adjust and scale. In this report, we analyze two real cases, one in pediatrics and one for end-of-life. We showcase how the BAIA framework can reduce moral distress on the care providers, harmonize differing perspectives, and enhance the quality and consistency of decisions. In highly emotional and critical scenarios, this advice from BAIA might bring a rational angle to advising surrogates and their families. Our next step is to apply this framework in real time to actual cases, validate outcomes, and establish baseline measures to assess its impact on moral distress and ethical residue.
